# Simultaneous fitting of genomic-BLUP and Bayes-C components in a genomic prediction model

**DOI:** 10.1186/s12711-017-0339-9

**Published:** 2017-08-24

**Authors:** Oscar O. M. Iheshiulor, John A. Woolliams, Morten Svendsen, Trygve Solberg, Theo H. E. Meuwissen

**Affiliations:** 1 Department of Animal and Aquacultural Sciences, Norwegian University of Life Sciences, PO Box 5003, 1432 Ås, Norway; 2 The Roslin Institute (Edinburgh), Royal (Dick) School of Veterinary Studies, University of Edinburgh, Midlothian, EH25 9RG Scotland, UK; 3 GENO SA, Holsegata 22, 2317 Hamar, Norway

## Abstract

**Background:**

The rapid adoption of genomic selection is due to two key factors: availability of both high-throughput dense genotyping and statistical methods to estimate and predict breeding values. The development of such methods is still ongoing and, so far, there is no consensus on the best approach. Currently, the linear and non-linear methods for genomic prediction (GP) are treated as distinct approaches. The aim of this study was to evaluate the implementation of an iterative method (called GBC) that incorporates aspects of both linear [genomic-best linear unbiased prediction (G-BLUP)] and non-linear (Bayes-C) methods for GP. The iterative nature of GBC makes it less computationally demanding similar to other non-Markov chain Monte Carlo (MCMC) approaches. However, as a Bayesian method, GBC differs from both MCMC- and non-MCMC-based methods by combining some aspects of G-BLUP and Bayes-C methods for GP. Its relative performance was compared to those of G-BLUP and Bayes-C.

**Methods:**

We used an imputed 50 K single-nucleotide polymorphism (SNP) dataset based on the Illumina Bovine50K BeadChip, which included 48,249 SNPs and 3244 records. Daughter yield deviations for somatic cell count, fat yield, milk yield, and protein yield were used as response variables.

**Results:**

GBC was frequently (marginally) superior to G-BLUP and Bayes-C in terms of prediction accuracy and was significantly better than G-BLUP only for fat yield. On average across the four traits, GBC yielded a 0.009 and 0.006 increase in prediction accuracy over G-BLUP and Bayes-C, respectively. Computationally, GBC was very much faster than Bayes-C and similar to G-BLUP.

**Conclusions:**

Our results show that incorporating some aspects of G-BLUP and Bayes-C in a single model can improve accuracy of GP over the commonly used method: G-BLUP. Generally, GBC did not statistically perform better than G-BLUP and Bayes-C, probably due to the close relationships between reference and validation individuals. Nevertheless, it is a flexible tool, in the sense, that it simultaneously incorporates some aspects of linear and non-linear models for GP, thereby exploiting family relationships while also accounting for linkage disequilibrium between SNPs and genes with large effects. The application of GBC in GP merits further exploration.

## Background

The rapid adoption of genomic selection (GS) is due to two key factors: (1) availability of high-throughput dense genotyping, and (2) availability of statistical methods to estimate and predict breeding values [1, 2]. The development of such methods is still ongoing and so far, there is no consensus on the best approach. The methods available for genomic prediction (GP), can be broadly classified into two groups: linear and non-linear methods [3]. Genomic-best linear unbiased prediction (G-BLUP) is a typical example of a linear method, while the Bayesian methods such as Bayes-(A/B/C/etc.), are non-linear methods and often implemented by Markov chain Monte Carlo (MCMC) algorithms. A major difference between the linear and non-linear methods lies in their prior assumptions about the effects of the single-nucleotide polymorphisms (SNPs), which have been reviewed in detail by Neves et al. [4] and De Los Campos et al. [5]. Currently, linear and non-linear methods are treated as distinct approaches, and results from most empirical studies show that they yield similar prediction accuracies. However, in contrast, simulation studies reported significant differences between linear and non-linear methods [6, 7], an issue which was resolved by Daetwyler et al. [3] who demonstrated that the number of QTL (quantitative trait loci) in relation to the structure of the genome was a major factor in this discrepancy.

G-BLUP is commonly used for routine genetic evaluations because of its simple and less computationally demanding nature. Since Bayesian methods are often implemented by using MCMC algorithms, they are time consuming and computationally demanding when they deal with large numbers of SNPs. Hence, they are rarely used in routine genetic evaluations although they can potentially pick up and use SNPs with large effects or the actual causative variants. The need to reduce computational demands, while maintaining the features of Bayesian methods, has led to the development of iterative methods (non-MCMC-based Bayesian methods) such as the VanRaden’s non-linear A/B [8], fastBayesB [9], MixP [10], or emBayesR [11] methods. These methods are iterative in nature hence computationally fast and yield prediction accuracies that are similar to those of MCMC-based Bayesian methods. However, they remain focused on exploiting linkage disequilibrium (LD) just as their MCMC-based counterparts.

GP uses two sources of information: genetic relationships among individuals and LD between SNPs and QTL [12, 13]. The emphasis put on these sources of information varies with the GP method used. G-BLUP through the genomic relationship matrix (**G)** exploits the relationship in a given population more comprehensively than the pedigree-based relationship matrix (**A)**, both by quantifying the variation in relationships between sibs and the historical relationships between individuals in the base generation of **A** [12, 14, 15]. However, compared to G-BLUP, non-linear methods can better exploit the LD information gained through mapping of QTL [12, 13]. Thus, methods that could exploit both genetic relationships and LD might help to increase prediction accuracy and the persistency of the accuracy across time and genetic distance.

Our aim was to develop an iterative method (referred to as GBC) that combines relationship information using the G-BLUP approach with information on the LD between QTL and neighboring SNPs using the Bayes-C [16] approach of GP. In a sense, GBC shares the Bayes-A property of including all SNPs in the prediction [7] but implies different prior assumptions on the effects. Given the importance of reducing computational demands when dealing with large numbers of SNPs, GBC follows the iterative approach of other non-MCMC-based methods but differs from both MCMC- and non-MCMC-based Bayesian methods by combining aspects of G-BLUP and Bayes-C methods for GP. We evaluated GBC using an imputed 50 K SNP chip dataset. Furthermore, predictions from GBC were compared to those from G-BLUP and Bayes-C, using real data from a population of genotyped bulls.

## Methods

### Phenotypes

Daughter yield deviations (DYD; [17]) on 3244 proven Norwegian Red bulls and their associated effective number of daughters (d_e_; i.e. weighted number of daughters for each bull) were obtained from GENO SA (http://www.geno.no). These were extracted from the routine genetic evaluations of 2013 for three production traits, fat yield (Fkg), milk yield (Mkg) and protein yield (Pkg), and a health indicator, somatic cell count (SCC). The DYD is an estimate of the average performance of each bull’s daughters, corrected for all fixed and non-genetic random effects of the daughters and genetic effects of the bulls’ mates [17]. The minimum d_e_ was 108 and the average d_e_ was 177 with a standard deviation of ~31. The reliabilities of the DYD were calculated following Fikse and Banos [18] as $r_{DYD}^{2} = d_{e} /\left( {d_{e} + K} \right),$ where *K* = (4 − *h* ^2^) and *h* ^2^ is the heritability of the trait used in the evaluations. The parameters used for each trait and average reliabilities for each trait are in Table 1. The average reliability between bulls ranged from 0.858 for SCC to 0.927 for Mkg.

**Table 1:** Heritability (h^2^) and average reliability (${\text{r}}_{\text{DYD}}^{2}$) of daughter yield deviations for the 3244 bulls

Trait	*h* ^2^	$r_{DYD}^{2}$
Somatic cell count (SCC)	0.136	0.858
Fat yield (Fkg)	0.213	0.906
Milk yield (Mkg)	0.277	0.927
Protein yield (Pkg)	0.235	0.915

$r_{DYD}^{2} = d_{e} /\left( {d_{e} + K} \right),$ where *d*_*e*_ is the effective number of daughters and *K* = (4 − *h* ^2^)/*h* ^2^

### Genotypes

Genotyping data were also provided by Geno SA for these bulls. Bulls were previously genotyped with different SNP chips: 2450 bulls with the 25 K Affymetrix chip (Affymetrix Inc., Santa Clara, CA), 1650 were genotyped with the Illumina Bovine50K BeadChip (Illumina Inc., San Diego, CA), and 856 were genotyped with both.

Quality control was carried out by CIGENE (http://www.cigene.no) and is described in detail by Solberg et al. [19]. Briefly, quality control was carried out post-genotyping within each set of SNP chip data so that animals with an individual call rate lower than 97% and SNPs with a call rate lower than 25% were removed. Pedigree relationships between parent and offspring were set to missing if they exceeded a Mendelian error threshold of 1%; following this, SNPs with an overall Mendelian error rate higher than 2.5% were deleted; and, for parent–offspring pairs with Mendelian errors less than 1%, SNP genotypes that were flagged as errors were set to missing. Finally, SNPs with a minor allele frequency lower than 0.05 were discarded.

We used genotype imputation to obtain ~50 K SNP genotypes. The genotypes of bulls obtained with the 25 K Affymetrix chip were imputed to the SNP density of the Illumina Bovine50K BeadChip. Genotype imputation was performed by CIGENE (http://www.cigene.no) using Beagle v3.3.1 [20] and other in-house developed software as described by Solberg et al. [19]. Following these procedures, the data contained 48,249 SNPs on 3244 bulls. SNPs that were not mapped to the bovine reference genome assembly UMD 3.1 [21] and those on the X chromosome were not included in the analyses.

### Reference and validation sets

Bulls were divided into reference and validation sets following a standard animal breeding selection scheme, so that the validation dataset consisted of the 124 youngest sires born between January 1st 2007 and December 31st 2008 with a minimum of 100 actual daughters. The reference set included bulls born between 1964 and 2005 with all performance records contributing to the DYD collected before January 1st 2007, for a total of 3091 bulls. To check relationships between reference and validation sets following Clark et al. [22] and Daetwyler et al. [23], four measures of genomic relatedness were calculated from the genomic relationship according to VanRaden’s method 1 [8]. For each bull these measures were: (1) the mean relationship with the reference population (meanRel); (2) the maximum relationship (Relmax); (3) the mean of the five largest absolute relationships (Rel5); and (4) the mean of the ten largest absolute relationships (Rel10).

### Genomic prediction methods and data analysis

Three methods were implemented for GP: G-BLUP, Bayes-C, and GBC. Genetic and error variances used in the analyses were estimated from the dataset using ASReml v3.0 [24].

#### G-BLUP

The G-BLUP model [7, 8] used to predict genomic estimated breeding values (GEBV) was as follows:

1 $${\mathbf{y}} = \mathbf{1}\mu + {\mathbf{Zg}} + {\mathbf{e}},$$

where **y** is a vector of DYD for the reference set; **1** is a vector of ones; *μ* is the overall mean; **Z** is a design matrix that relates the records to genomic values; **g** is a vector of genomic values assumed to follow a multivariate normal distribution *MVN* ~ (0, $\sigma_{g}^{2} {\mathbf{G}}$), where **G** is the genomic relationship matrix and $\sigma_{g}^{2}$ is the genetic variance; and **e** is the vector of residuals assumed to follow a multivariate normal distribution *MVN* ~ (0, $\sigma_{e}^{2} {\mathbf{I}}$). **G** was calculated, following VanRaden’s method 1 [8] using all bulls, as ${\mathbf{G}} = {\mathbf{MM}}^{\prime } /2\sum p_{j} \left( {1 - p_{j} } \right)$, and *M*_*ij*_ = *x*_*ij*_ − 2*p*_*j*_, where *x*_*ij*_ is the genotype of bull *i* for SNP *j*, with *x*_*ij*_ = 0, 1 or 2 for the reference homozygote, heterozygote and alternative homozygote, respectively, and *p*_*j*_ is the allele frequency of the alternative allele of SNP *j* for all bulls.

#### Bayes-C

Bayes-C, a sub-model of GBC (i.e. where the variance explained by the GBLUP term in GBC is set to zero), was also independently evaluated so that the relative performance of both approaches can be compared. Bayes-C assumes that a fraction (1 − *π*) of the SNPs has zero effects and that the distribution of the effects for the other fraction (*π*) is normal [16]. Thus, the model of analysis for Bayes-C is:

2 $${\mathbf{y}} = \textbf{1}\mu + {\mathbf{ZMQq}} + {\mathbf{e}},$$

where **M** is the design matrix of scaled SNP genotypes as in the calculation of **G** above; **Q** is a diagonal matrix with indicators on the diagonal that are 1 if the SNP has an effect (with prior probability *π*) and 0 if it has no such effect (with prior probability (1 − *π*); **q** is a vector of SNP effects (*q*_*j*_) assumed to be normally distributed, i.e. $q_{j} \sim\,N\left( {0,\sigma_{q}^{2} } \right)$ with probability *π* and 0 otherwise. All other model elements are defined as previously. The *π* values used were estimated from the dataset via a search between 1% and then 5 to 30% in increments of 5% to obtain the optimal *π* values. The GEBV for the validation animals was calculated as ${\mathbf{M}}_{v} {\hat{\mathbf{q}}}$ where **M**_*v*_ describes the scaled genotypes for each bull in the validation set, and ${\hat{\mathbf{q}}}$ is the posterior mean of the SNP effects. Bayes-C analyses were performed using the GS3 software [25]. The number of iterations was 20,000 with a burn-in of 2000 and a thinning interval of 100. Using 50,000 or 100,000 iterations with a burn-in of 10,000 or 20,000 had no impact on the accuracy of prediction but increased computing time.

#### GBC

This method fits a Bayes-C model [16] simultaneously with an effect due to background genes following a GBLUP model. This was achieved by using the iterative conditional expectation (ICE) algorithm [9], to which was added a correction for the uncertainty of the other effects of SNPs when deciding whether SNP *j* has an effect or not as described by Wang et al. [11]. The ICE algorithm uses the expectation/mean instead of the posterior mode, mainly because the posterior distribution is often bimodal, and when both modes are about equally high, the mode of the distribution is rather an arbitrary choice. The model of analysis used by GBC is:

3 $${\mathbf{y}} = \textbf{1}\mu + {\mathbf{ZMQq}} + {\mathbf{Zg}} + {\mathbf{e}},$$

where **g** is a vector of residual breeding values with distributional assumptions as described above for G-BLUP. All other elements of the model are defined as previously. The *π* values were estimated from the dataset via a search between 1% and then 5 to 30% in increments of 5% to obtain the optimal *π* values.

The G-BLUP term was implemented as described in the section on G-BLUP, but here, it is called the residual breeding value because it represents the breeding value after the SNPs with the largest effects have been fitted through the Bayes-C term. In the Bayes-C term, the SNPs with a large effect were assumed to have a variance of 0.001 $\sigma_{g}^{2}$ as implemented here. Optimal *π* values and the fraction of genetic variance explained by the SNPs with a large effect in GBC are assessed by cross-validation.

#### Posterior probabilities of SNPs with a large effect in GBC

The posterior probability that a SNP *j* has a large effect is calculated from:

$$PostProb\left( {Q_{jj} = 1} \right) = \frac{{PPR_{j} * LR_{j} }}{{PPR_{j} *LR_{j} + 1}},$$

where *PPR*_*j*_ is the prior-probability-ratio (=*π*(1 − *π*)); and *LR*_*j*_ is the likelihood ratio that SNP *j* has a large effect. The log(*LR*_*j*_) equals the log-likelihood of a model with versus without the effect of SNP *j* (see Appendix for a derivation):

$$\begin{aligned} \log \left( {LR_{j} } \right) = & \,\frac{1}{2}\log \left( \lambda \right) - \frac{1}{2}\log \left( {m_{j}^{\prime } m_{j} + \lambda } \right) \\ & + \frac{1}{2}(y^{*\prime } m_{j} m_{j}^{\prime } y^{*} + m_{j}^{\prime } PEVm_{j} )\sigma_{e}^{ - 2} /\left( {m_{j}^{\prime } m_{j} + \lambda } \right), \\ \end{aligned}$$

where $\lambda = \sigma_{e}^{2} /\sigma_{q}^{2}$; *m*_*j*_ are the scaled genotypes of SNP *j* for animals with records; *y* ^*^ are the records corrected for all other effects in the model except that of SNP *j*; *PEV* is the prediction error variance matrix of the G-BLUP model; and the $m_{j}^{\prime } PEVm_{j} /\left( {m_{j}^{\prime } m_{j} + \lambda } \right)$ term corrects for the uncertainty about the other genetic effects in the model [11].

The effect of SNP *j* now becomes:

$$\hat{q}_{j} = PostProb\left( {Q_{jj} = 1} \right)*m_{j}^{\prime } y^{*} /\left( {m_{j}^{\prime } m_{j} + \lambda } \right),$$

where the $m_{j}^{\prime } y^{*} /\left( {m_{j}^{\prime } m_{j} + \lambda } \right)$ term equals the BLUP solution of the SNP effect when it has a large effect.

### Predictive ability

The primary criterion for evaluating predictive ability was the accuracy of the predictions (*r*), calculated as the correlation between GEBV and DYD, divided by the square root of the average reliability of the DYD for the trait $\left( {\sqrt {r_{DYD}^{2} } } \right)$. The bias of predictions was calculated as the unweighted regression of DYD on the predicted values, where a regression coefficient of 1 denotes no bias, less than 1 implies that the spread of the GEBV is too large, and more than 1 implies their spread is too small.

Standard errors of the prediction accuracies and the regression coefficients on the DYD were computed using a custom bootstrapping R-script in R software [26]. The bootstrap procedure involved sampling with replacement of the GEBV 10,000 times. For each bootstrap sample, pairs of GEBV-DYD of an animal in the validation population are sampled with replacement, i.e. the connection between a specific GEBV and DYD is maintained in this sampling process. The resulting GEBV were correlated to the DYD, and standard errors were computed from the 10,000 bootstrap estimates of accuracy and bias. A Hotelling–Williams test [27] for dependent correlations was used to determine whether differences between the validation correlations using alternative methods were statistically significant.

## Results

### Genomic relatedness between validation and reference individuals

Table 2 shows the average genomic relatedness between reference individuals and between validation and reference individuals. Overall meanRel was equal to 0.03, while estimated Relmax between the validation and reference population was ~0.5, which suggests that nearly all the bulls in the validation population were closely related to the reference population (i.e. their sire is in the reference population). For Rel5 and Rel10, genomic relatedness estimates of 0.29 and 0.24, respectively, were obtained.

**Table 2:** Average of four measures of genomic relatedness

Relatedness	meanRel	Relmax	Rel5	Rel10
Within reference	0.03 (0.01)	0.49 (0.04)	0.34 (0.05)	0.30 (0.05)
Between validation and reference	0.03 (0.00)	0.48 (0.09)	0.29 (0.05)	0.24 (0.05)

Standard deviations are in parentheses

Here, meanRel is the average relationship $(1/N_{P} )\sum\nolimits_{j = 1}^{{N_{p} }} r el\left( {i,j} \right),$ where *N*_*P*_ is the number of individuals in the reference population, *rel*(*i*, *j*) is the relationship between validation *i* and reference individual *j*; Relmax is the maximum (*rel*(*i*, *j*))for individual *i* over all reference individuals *j*; Rel5 is $(1/5)\sum\nolimits_{j = 1}^{{N_{p} }} {x_{ij} } rel\left( {i,j} \right),$ where *x*_*ij*_ = 1 if *j* is among the top 5 (*i*, *j*) for individual *i* and Rel10 is the extension to the top 10 relationships for *i*

### Prediction methods

Table 3 shows the accuracies of predictions using alternative prediction methods. Accuracies across the four traits ranged from 0.602 to 0.716 for G-BLUP, from 0.604 to 0.733 for Bayes-C, and from 0.607 to 0.731 for GBC. The highest accuracy was found for Fkg across the three methods. Apart from the trait Fkg for which GBC resulted in a statistically significant higher accuracy than G-BLUP using the Hotelling–Williams test (P < 0.05), we observed that, although not significant, in most cases the accuracies obtained with GBC were higher than with G-BLUP and Bayes-C. Generally, on average across the four traits, G-BLUP yielded the lowest prediction accuracy while GBC yielded the highest prediction accuracy. GBC yielded a 0.009 and 0.006 increase in prediction accuracy over G-BLUP and Bayes-C, respectively. The regression coefficients (Table 4) ranged from 0.881 to 0.956 for SCC, from 1.259 to 1.326 for Fkg, from 1.435 to 1.530 for Mkg, and from 1.410 to 1.506 for Pkg. Regression coefficients differed slightly across methods.

**Table 3:** Accuracy (SE) of the predicted values for the youngest sires based on the different prediction methods

Trait_(π)_	G-BLUP	Bayes-C	GBC
SCC _(20%, 20%,)_	0.602 (0.066)	0.604 (0.064)	0.607 (0.065)
Fkg _(10%, 10%,)_	0.716 (0.049)	0.733 (0.042)	0.731 (0.047)
Mkg _(10%, 10%,)_	0.705 (0.051)	0.701 (0.050)	0.719 (0.048)
Pkg _(10%, 1%,)_	0.695 (0.053)	0.689 (0.050)	0.696 (0.051)
Average	0.679	0.682	0.688

$${\text{Accuracy }} = \frac{{corr\left( {DYD,GEBV} \right)}}{{\sqrt {r_{DYD}^{2} } }}$$

SE: standard errors computed from 10,000 bootstrap samples

G-BLUP: genomic BLUP using genomic-based relationship matrix; Bayes-C: a non-linear method that fits zero effects and normal distributions of effects for SNPs; GBC: an iterative method that fits a G-BLUP next to SNP effects with a Bayes-C prior

*SCC*, somatic cell count;* Fkg*, fat yield;* Mkg*, milk yield;* Pkg*, protein yield

π refers to the optimal π values (i.e. proportion of SNP having large effects) when using Bayes-C and GBC

**Table 4:** Bias (SE) of the predicted values for the youngest sires based on the different prediction methods

Trait	G-BLUP	Bayes-C	GBC
SCC	0.881 (0.111)	0.956 (0.120)	0.881 (0.109)
Fkg	1.275 (0.120)	1.326 (0.131)	1.259 (0.113)
Mkg	1.530 (0.146)	1.435 (0.136)	1.459 (0.136)
Pkg	1.506 (0.157)	1.410 (0.149)	1.461 (0.100)

Bias: measured as the regression of daughter yield deviation on the predicted values

SE: standard errors computed from 10,000 bootstrap samples

G-BLUP: genomic BLUP using genomic-based relationship matrix; Bayes-C: a non-linear method that fits zero effects and normal distributions of effects for SNPs; GBC: an iterative method that fits a G-BLUP next to SNP effects with a Bayes-C prior

*SCC*, somatic cell count;* Fkg*, fat yield,* Mkg*, milk yield;* Pkg*, protein yield

### Effects of SNPs: Bayes-C and GBC

**Fig. 1:**
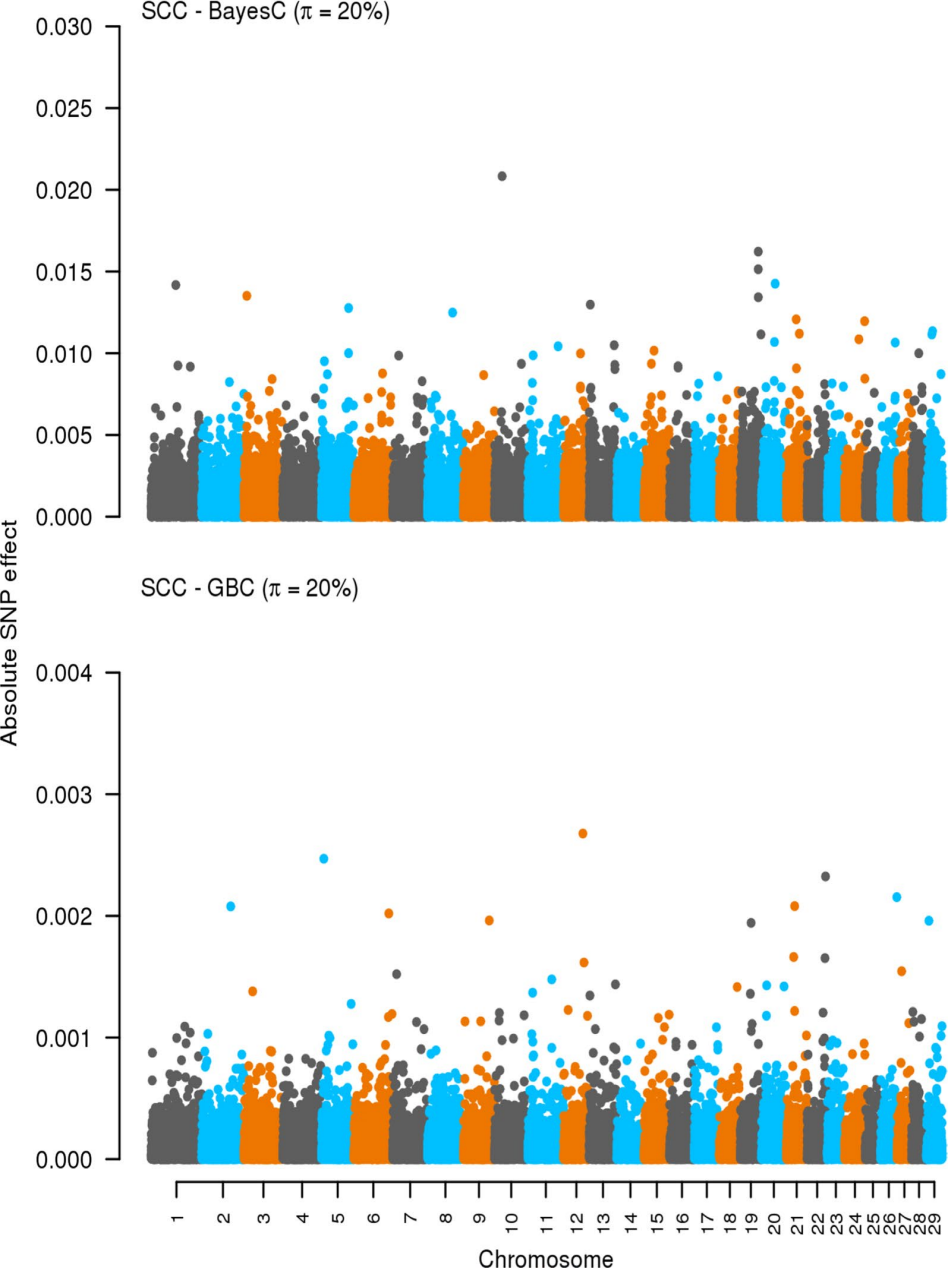
Effects of SNPs estimated by using Bayes-C and GBC for somatic cell count (SCC). The absolute values of the estimates of the effects of SNPs are on the *y axis*. The *X axis* is ordered by chromosomes from 1 to 29. *π* refers to the optimal *π* value when using Bayes-C and GBC. Absolute values were standardized by $\sqrt {\sigma_{g}^2}$. Standardization was only for plotting purpose

**Fig. 2:**
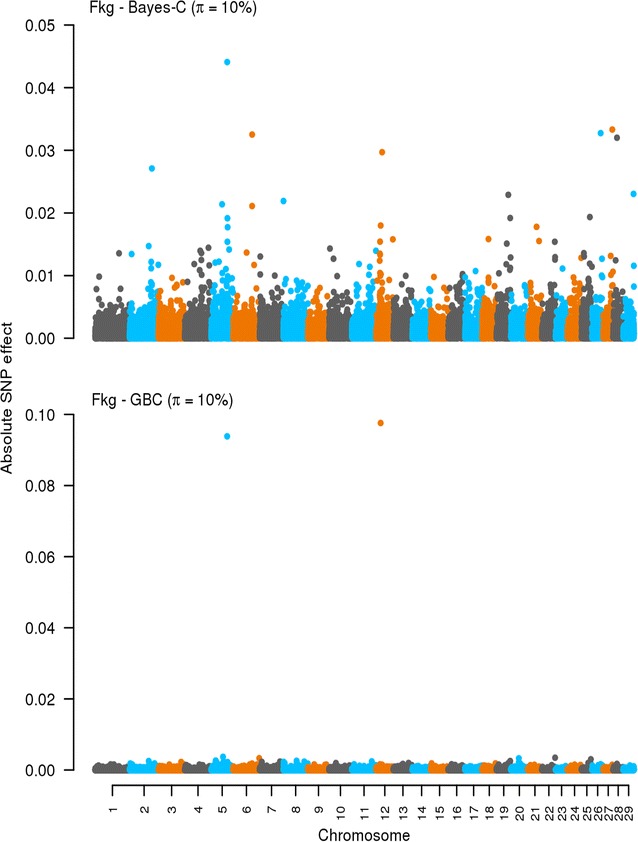
Effects of SNPs estimated by using Bayes-C and GBC for fat yield (Fkg). The absolute values of the estimates of the effects of SNPs are on the *y axis*. The *X axis* is ordered by chromosomes from 1 to 29. *π* refers to the optimal *π* value when using Bayes-C and GBC. Absolute values were standardized by $\sqrt {\sigma_{g}^2}$. Standardization was only for plotting purpose

**Fig. 3:**
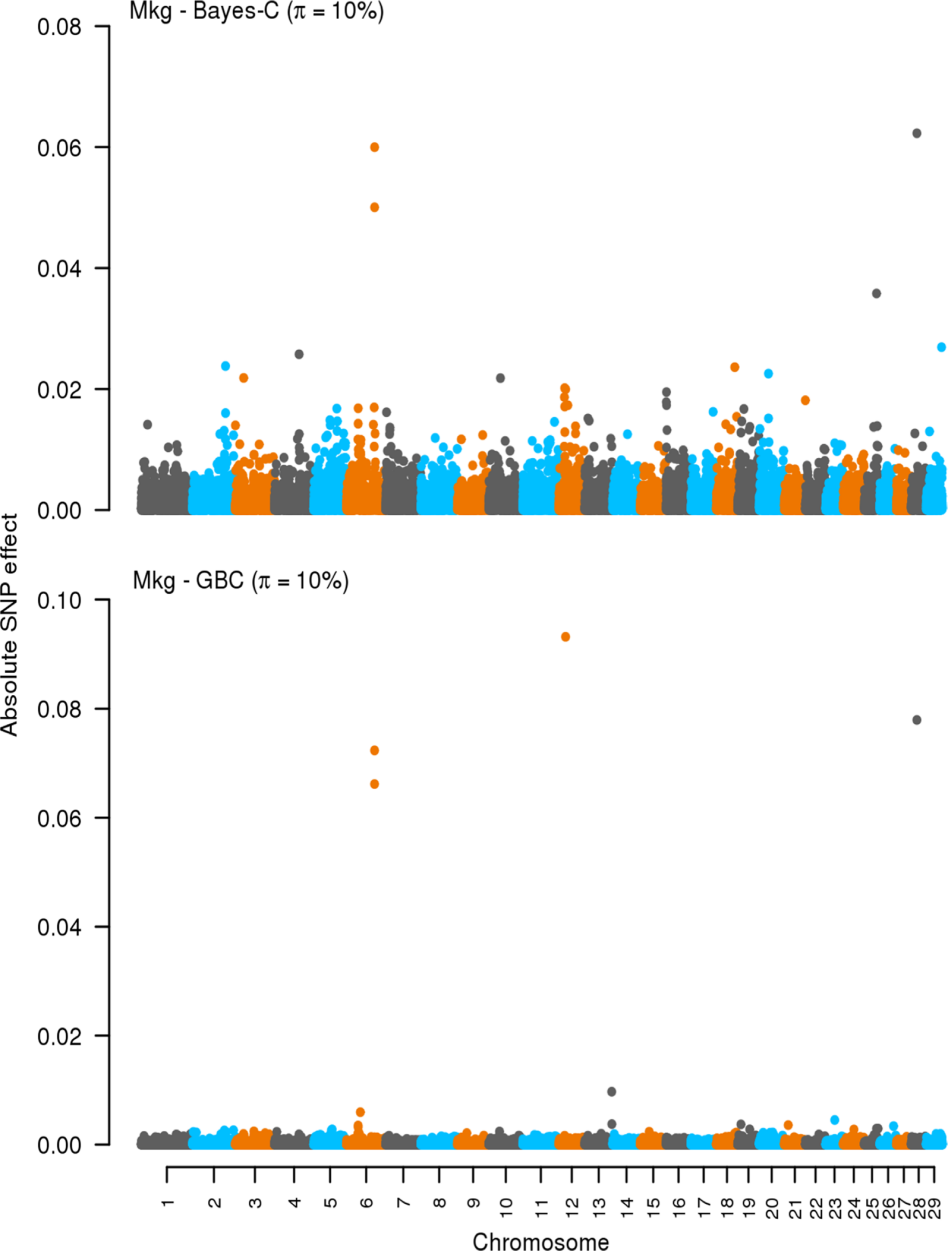
Effects of SNPs estimated by using Bayes-C and GBC for milk yield (Mkg). The absolute values of the estimates of the effects of SNPs are on the *y axis*. The *X axis* is ordered by chromosomes from 1 to 29. *π* refers to the optimal *π* value when using Bayes-C and GBC. Absolute values were standardized by $\sqrt {\sigma_{g}^2}$. Standardization was only for plotting purpose

**Fig. 4:**
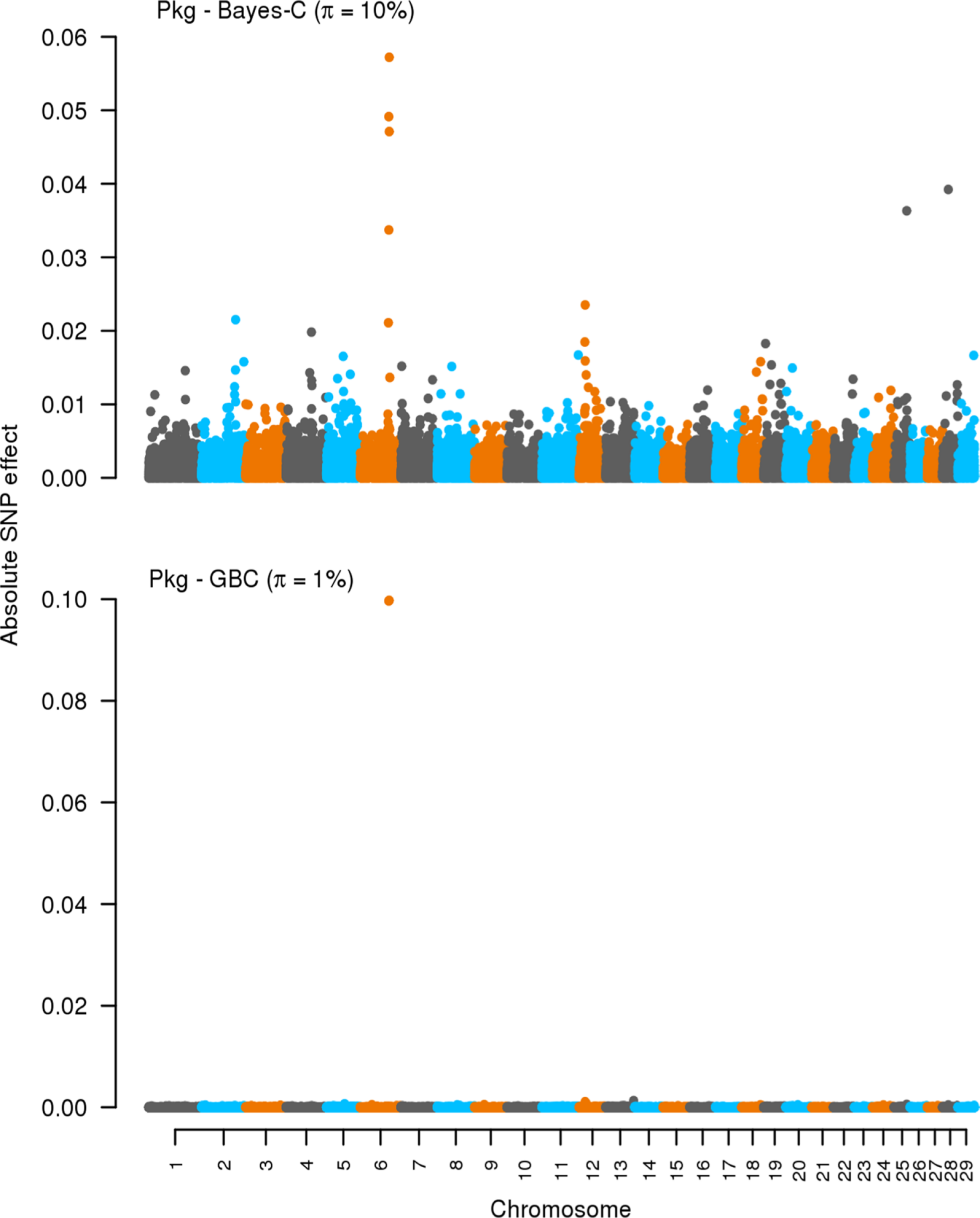
Effects of SNPs estimated by using Bayes-C and GBC for protein yield (Pkg). The absolute values of the estimates of the effects of SNPs are on the *y axis*. The *X axis* is ordered by chromosomes from 1 to 29. *π* refers to the optimal *π* value when using Bayes-C and GBC. Absolute values were standardized by $\sqrt {\sigma_{g}^2}$. Standardization was only for plotting purpose

The effects of SNPs estimated by Bayes-C and GBC are in Figs. 1, 2, 3 and 4. For Fkg, GBC picked up two SNPs with a large effect on chromosomes 5 and 12. The effects of the other SNPs were substantially shrunk towards 0. With Bayes-C, the same SNPs were observed to have large effects but several other SNPs with small to moderate effects were also found. For Mkg, we observed a similar trend, i.e. GBC identified SNPs on chromosomes 6, 12, and 28 with a large effect while Bayes-C also identified SNPs on chromosomes 6 and 12 as well as other SNPs. Chromosome 6 was also identified by both methods as a region that carries SNPs with a large effect on Pkg. In the case of SCC, there were many SNPs with (very) small effects across the genome as indicated by both methods especially with GBC.

### Computing time and memory usage

Table 5 shows the computing time and memory usage for each method. With an Intel(R) Xeon(R) CPU E5-2670 0 @ 2.60 GHz, G-BLUP took on average 2.51 min with average memory usage of about 2197 MB to complete the analysis, Bayes-C took on average 1.10 h with average memory usage of about 1296 MB, while GBC took on average 4.2 min with average memory usage of 2474 MB. Generally, across the four traits studied, G-BLUP was fastest, followed closely by GBC in terms of computing time while in terms of memory usage Bayes-C used less memory compared to G-BLUP and GBC.

**Table 5:** Computing time of the different prediction methods for each trait

Method	SCC	Fkg	Mkg	Pkg
G-BLUP	00:03:24 (2233.492 MB)	00:02:18 (2233.492 MB)	00:02:31 (2159.716 MB)	00:02:31 (2159.716 MB)
Bayes-C	01:04:06 (1296.312 MB)	01:10:32 (1296.312 MB)	01:14:41 (1296.312 MB)	01:10:36 (1296.312 MB)
GBC	00:03:04 (2474.432 MB)	00:04:51 (2474.436 MB)	00:05:11 (2474.436 MB)	00:04:14 (2474.436 MB)

Memory usage is in parentheses

G-BLUP: genomic BLUP using genomic-based relationship matrix; Bayes-C: a non-linear method that fits zero effects and normal distributions of effects for SNPs; GBC: an iterative method that fits a G-BLUP next to SNP effects with a Bayes-C prior

*SCC* somatic cell count;* Fkg*, fat yield,* Mkg*, milk yield;* Pkg*, protein yield

## Discussion

GP uses mainly two sources of information: genetic relationships between individuals and LD between SNPs and QTL [12, 13]. The contribution of both information sources to prediction in a given population can vary across generations with relationships decaying across generations while LD may remains fairly persistent [12, 13]. Currently, these sources are included separately in the linear (i.e. G-BLUP) and non-linear (i.e. Bayesians) GP methods. While G-BLUP tries to exploit relationships maximally, the Bayes-(A/B/C/etc.) methods try to use LD between individual SNPs and genes maximally. To take advantage of both methods as well as to maintain short computing times, we developed and evaluated an iterative GP method, i.e. GBC that combines relationship information using the G-BLUP approach with information on LD between QTL and neighboring SNPs using the Bayes-C approach. Comparisons were made with the commonly used G-BLUP, which does not select SNPs, and Bayes-C, a non-linear method that assumes zero effects for a fraction of the SNPs and a normal distribution of the effects for the other fraction. Our results show that simultaneously fitting a GBLUP and a Bayes-C term can improve accuracy over G-BLUP and Bayes-C, alone. In terms of computational speed, GBC was much faster than a MCMC-based version of Bayes-C but used more memory compared to GBLUP and Bayes-C.

### Prediction methods

In this study, we compared our new method GBC to two existing GP methods: G-BLUP and Bayes-C. Generally, on average across the four traits, GBC yielded a 0.009 and 0.006 increase in prediction accuracy over G-BLUP and Bayes-C, respectively. With GBC, we anticipated that, by fitting a residual SNP term in addition to Bayes-C SNP effects, both models would complement each other: the G-BLUP term mainly picking up effects that could be explained by linkage analysis [12] and the Bayes-C term picking up tight LD between SNPs and genes. Consequently, we expected GBC to result in a higher accuracy of GP. Although the results agreed with this expectation, differences were small and were generally not statistically significant.

The GBC method has some similarity with Bayes-A [7], i.e. both methods fit all SNPs in the model while differentiating between SNPs with a large variance and SNPs with a small variance. Habier et al. [16] observed that Bayes-A performed marginally better than G-BLUP and Bayes-C with real data. GBC performed always marginally better than G-BLUP and Bayes-C, except for Fkg where it performed significantly better than G-BLUP. A possible explanation could be that the modeled LD blocks that surround the major genes were large and were also reasonably well captured by G-BLUP [3]. In addition, as shown in Table 2, the relationships between the animals in the validation set and those in the reference set were generally high in our dataset. In such a case, the performance of GBC is only marginally better than that of G-BLUP and Bayes-C modeled independently. This suggests that GBC works well if the level of relationships is high. If there are no relationships or if relationships decay across generations while LD remains fairly persistent, GBC also has the potential to yield more persistent accuracies across generations since it models both information sources simultaneously. Practically, the number of QTL or major genes that underlie a trait remains largely unknown and so is the variance explained by the genes with large effects. Examples of genes with a large effect are reported in the literature, e.g. the *diacylglycerol O-acyltransferase 1* (*DGAT1*) gene involved in fat percentage in dairy cattle [28]. The GBC model assumes that some genes with a large effect can be detected based on LD while the effects of the background genes are predicted by genomic relationships, which seems to be mainly true for Fkg.

GBC, as mentioned earlier, simultaneously fits Bayes-C and G-BLUP components. This means that all SNPs are included twice in the model, first in the Bayes-C term, and second in the G-BLUP term. The G-BLUP term includes all the SNPs to explain genomic relationships between the animals, whereas the Bayes-C term answers the question whether a SNP might explain more variance than expected based on its contribution to genomic relationships (where all SNPs contribute equally). Thus, GBC opens the opportunity for SNPs with extra-large effects to be included twice in the model and thereby increasing their weight in the GP.

The regression coefficients in Table 4 are a measure of bias of the GEBV predictions. Except for the trait SCC for which the regression coefficients were lower than 1, they were above 1 for the other three traits across all methods. This implies that, for the production traits, the variance of GEBV was deflated while for SCC, it was inflated. Since, in Norwegian Red cattle, selection pressure against directly recorded mastitis is strong and mastitis is quite highly correlated to SCC, biased GP is expected for SCC (a bivariate analysis that would fit both mastitis and SCC might avoid such bias). For the production traits (Fkg, Mkg and Pkg), all investigated methods also yielded biased GEBV, which is probably due to these 124 validation bulls being under strong selection for these traits. Considering that the regression coefficients from the pedigree-BLUP (result not shown) showed similar biases, one may attribute the biases to intrinsic aspects of the data such as selection.

### Effects of SNPs: Bayes-C and GBC

A key difference between Bayes-C and GBC lies in how they estimate and deal with the effects of SNPs. Bayes-C assumes a priori zero effects for a fraction (1 − *π*) of the SNPs and a normal distribution of effects for the other fraction (*π*) [16]. GBC fits a Bayes-C like prior for the SNPs with large effects assuming that an estimated fraction *π* of the SNPs have a large effect with a variance of 0.001 $\sigma_{g}^{2}$ (this proportion can differ across traits) and then it fits a G-BLUP component for all SNPs. With GBC, all SNPs have an estimated effect, thus, in a sense, GBC methods share the Bayes-A property of including all SNPs in the prediction [7] but their prior assumptions about SNP effects differ. As shown in Figs. 1, 2, 3 and 4, the methods behaved differently in terms of number of SNPs with effects and their magnitude. However, interestingly for the production traits, both methods found common SNPs with large effects on chromosomes 5, 6, and 12. We did not try to identify candidate genes in these regions, as this was outside the scope of our study. Nevertheless, several genome-wide association studies (GWAS) have reported that these chromosomes harbor QTL that affect production traits in dairy cattle [29–32]. In the case of SCC, both methods showed no clear pattern with many SNPs having very small effects.

There was a general tendency that GBC allocated large effects to (very) few SNPs while Bayes-C identified many more SNPs with moderate to large effects. This implies that, with GBC, only SNPs in high LD with the QTL tend to pick up the genes with major effects while the others are treated as residual SNP effects. Bayes-C also needs to capture SNP genetic relationships by fitting SNPs with large effects, and thus needs to fit more SNPs. Therefore, the observed differences in prediction accuracies between both methods are a reflection of how genomic regions with large and small effects are treated. The ability of GBC to not neglect any SNP effect may explain why it tended towards higher accuracies than Bayes-C. It seems that, the GBC method is also very precise in pointing towards QTL locations. This could be due to GBC showing some similarity to GWAS methods in which a single SNP and a G-BLUP term are fitted, with the G-BLUP term correcting the QTL signal for family relationships. Although the prior distribution of the effects of SNPs and the actual proportion of variance they explain remain unknown, the results of this study indicate that the assumed prior distribution for the effects of SNPs alongside the proportion of variance they explain in GBC tends to yield somewhat higher accuracy than the assumptions underlying Bayes-C.

### Impact of the assumed variance for SNPs with a large effect in GBC

In this study, we assumed that, across the four traits studied, the SNPs with a large effect explained 0.1% of the genetic variance in GBC. This corresponds to the genetic variance explained by the third distribution in Bayes-R [33]. Bayes-R assumes that the effects of SNPs are derived from a mixture of four different normal distributions, each explaining 0, 0.01, 0.1, or 1% of the genetic variance, respectively. In our study, we set the genetic variance explained by SNPs to 0.1% because we considered that it was an intermediate value between that of genes with a small or a large effect. In addition, we did not want a situation where (very) few SNPs with a large effect explained a larger proportion of the genetic variance since most traits in livestock are polygenic. However, the proportion of genetic variance explained by the SNPs with a large effect might differ across traits. To investigate the impact of alternative assumptions on the variance explained for genes with a large effect, we also investigated a situation in which genes with a large effect explained 1% of the genetic variance. Assuming that SNPs with a large effect in GBC explained 1% of the genetic variance led to a 0.002 and 0.007 increase in prediction accuracy for Fkg and Mkg, respectively (result not shown), whereas for SCC and Pkg, it led to a 0.003 and 0.001 decrease in prediction accuracy, respectively. These results suggest that, in GBC, the optimal proportion of the genetic variance explained by SNPs with a large effect in GBC varies with traits. However, as also shown by the results, deviation from a 0.1% genetic variance explained by SNPs with a large effect seems to have little impact on prediction accuracy in GBC.

### GBC and other non-MCMC-based Bayesian methods

On the one hand, GBC shares some similarity with other non-MCMC-based Bayesian methods in the sense that it uses an iterative approach. A key advantage of the iterative methods (non-MCMC-based methods) over MCMC-based methods is their shorter computing time. Non-MCMC-based Bayesian methods such as fastBayesB [9], MixP [10], emBayesR [11] or VanRaden’s non-linear method [8] among others are computationally several orders faster than their MCMC counterparts. This is because generally, non-MCMC-based methods require much fewer iterations compared to MCMC-based methods. In agreement with the aforementioned studies, our results demonstrated the faster computing time of GBC compared to MCMC implementations of e.g. Bayes-C (Table 5). On the other hand, GBC differs from the aforementioned non-MCMC-based methods in that it simultaneously incorporates aspects of G-BLUP and Bayes-C methods for GP and thereby making it flexible for exploiting information of genomic data. In addition, unlike most other non-MCMC-based methods, GBC adds a correction for the uncertainty of other SNP effects when deciding whether a particular SNP has an effect or not as recommended by Wang et al. [11]. Not accounting for these uncertainties could result in a decline of about 8 to 9% in accuracy of prediction as demonstrated by Wang et al. [11].

## Conclusions

We introduced and evaluated the GBC method for GP, which simultaneously fits G-BLUP and Bayes-C terms. The method was evaluated by using imputed 50 K SNP datasets and its relative performance was compared to G-BLUP and Bayes-C. GBC showed marginal advantages over G-BLUP and Bayes-C for most of the traits in terms of prediction accuracy. For Fkg, GBC performed significantly better than G-BLUP, which agrees with the fact that Fkg is controlled by a few genes with a large effect. Overall in our study, statistically, GBC did not significantly outperform G-BLUP and Bayes-C probably due to a high level of relationship between reference and validation individuals. However, it is a flexible tool in the sense that it simultaneously incorporates some aspects of both linear and non-linear models for GP, thereby exploiting family relationships while also accounting for LD between SNPs and genes with a large effect. Computationally, GBC was much faster than Bayes-C with a computational speed that is comparable to that of G-BLUP. The application of GBC in GP merits further exploration.

## Appendix: Log likelihood ratio of a SNP having a normally distributed effect versus no effect

The general form of the multivariate normal probability density is:

$$P\left( {{\mathbf{y}} |\mu ,{\mathbf{V}}} \right) \propto \left| {\mathbf{V}} \right|^{{ - \frac{1}{2}}} { \exp }\left[ { - \frac{1}{2}({\mathbf{y}} - \mu )^{\prime } {\mathbf{V}}^{ - 1} \left( {{\mathbf{y}} - \mu } \right)} \right],$$

where **y** is a vector of multivariate normally distributed variables with mean *μ* and (co)variance matrix **V**.

First, we assume a model without a SNP effect and assume that ${\mathbf{V}} = {\mathbf{I}}\sigma_{e}^{2}$, and *μ* = 0 for simplicity (assuming the actual data are corrected for all other effects in the model except for the putative SNP effect). The log-likelihood of this null-model becomes:

$${\text{Log}}L\left( 0 \right) = - \frac{1}{2}n\log \left( {\sigma_{e}^{2} } \right) - \frac{1}{2}\frac{{y^{\prime } y}}{{\sigma_{e}^{2} }}.$$

For the alternative model with a SNP effect, we have ${\mathbf{V}} = \left( {{\mathbf{I}}\sigma_{e}^{2} + {\mathbf{mm}}^{\prime } \sigma_{q}^{2} } \right)$, where **m** is a vector of SNP genotypes of the animals with records, and $\sigma_{q}^{2}$ is the variance of the SNP effect. The log|**V**|^−1/2^ term becomes:

$$\begin{aligned} - \frac{1}{2}\log \left| {\mathbf{V}} \right| & = - \frac{1}{2}\log \left[ {(\sigma_{e}^{2} )^{n} \left( {\sigma_{q}^{2} } \right)\left( {\frac{{{\mathbf{m}}^{\prime } {\mathbf{m}}}}{{\sigma_{e}^{2} }} + \frac{1}{{\sigma_{q}^{2} }}} \right)} \right] \\ & = - \frac{1}{2}n\log \left( {\sigma_{e}^{2} } \right) + \frac{1}{2}\log \left( \lambda \right) - \frac{1}{2}{ \log }\left( {{\mathbf{m}}^{\prime } {\mathbf{m}} + \lambda } \right), \\ \end{aligned}$$

where $$\lambda = {\raise0.7ex\hbox{${\sigma_{e}^{2} }$} \!\mathord{\left/ {\vphantom {{\sigma_{e}^{2} } {\sigma_{q}^{2} }}}\right.\kern-0pt} \!\lower0.7ex\hbox{${\sigma_{q}^{2} }$}}$$.

Following Woodbury [34], the inverse of **V** can be written as:

$${\mathbf{V}}^{ - 1} = ({\mathbf{I}}\sigma_{e}^{2} + {\mathbf{mm}}^{\prime } \sigma_{q}^{2} )^{ - 1} = {\mathbf{I}}\sigma_{e}^{ - 2} - \frac{{\sigma_{e}^{ - 2} {\mathbf{mm}}^{\prime } }}{{\left( {{\mathbf{m}}^{\prime } {\mathbf{m}} + \lambda } \right)}} .$$

Such that **y**′**V** ^−1^ **y** becomes:

$${\mathbf{y}}^{\prime } {\mathbf{V}}^{ - 1} {\mathbf{y}} = \frac{{{\mathbf{y}}^{\prime } {\mathbf{y}}}}{{\sigma_{e}^{2} }} - \frac{{{\mathbf{y}}^{\prime } {\mathbf{mm}}^{\prime } {\mathbf{y}}}}{{\left( {{\mathbf{m}}^{\prime } {\mathbf{m}} + \lambda } \right)\sigma_{e}^{2} }}.$$

The log-likelihood of the alternative model with a SNP effect thus becomes:

$$\begin{aligned} {\text{Log}}L\left( a \right) = & \, - \frac{1}{2}n\log \left( {\sigma_{e}^{2} } \right) + \frac{1}{2}\log \left( \lambda \right) \\ & - \frac{1}{2}\log \left( {{\mathbf{m'm}} + \lambda } \right) - \frac{1}{2}\frac{{{\mathbf{y}}^{\prime } {\mathbf{y}}}}{{\sigma_{e}^{2} }} + \frac{1}{2}\frac{{{\mathbf{y}}^{\prime } {\mathbf{mm}}^{\prime } {\mathbf{y}}}}{{\left( {{\mathbf{m}}^{\prime } {\mathbf{m}} + \lambda } \right)\sigma_{e}^{2} }}. \\ \end{aligned}$$

Taking the log-likelihood ratio of the alternative to the null-model yields:

$$\begin{aligned} & {\text{Log}}L\left( a \right) - {\text{Log}}L\left( 0 \right) \\ & \quad = \frac{1}{2}\log \left( \lambda \right) - \frac{1}{2}\log \left( {{\mathbf{m}}^{\prime } {\mathbf{m}} + \lambda } \right) + \frac{1}{2}\frac{{{\mathbf{y}}^{\prime } {\mathbf{mm}}^{\prime } {\mathbf{y}}}}{{\left( {{\mathbf{m}}^{\prime } {\mathbf{m}} + \lambda } \right)\sigma_{e}^{2} }}. \\ \end{aligned}$$

Following Wang et al. [11], we account for the fact that the correction of the data **y** was not performed using the true value of all other effects in the model, but using estimates of these effects, which results in an estimate of **y** denoted by **y** ^*^. The variance of **y** ^*^ given the real data **y** is denoted by the prediction error covariance matrix PEV, which is assumed approximately equal to the PEV matrix from the G-BLUP model. Accounting for this uncertainty due to prediction error variances of **y** ^*^, the expectation of the **y**′**mm**′**y** term is:

$$\begin{aligned} E\left( {{\mathbf{y}}^{\prime } {\mathbf{mm}}^{\prime } {\mathbf{y}}} \right) & = {\mathbf{y}}^{*\prime } {\mathbf{mm}}^{\prime } {\mathbf{y}}^{*} + trace\left( {PEV{\mathbf{mm}}^{\prime } } \right) \\ & = {\mathbf{y}}^{*\prime } {\mathbf{mm}}^{\prime } {\mathbf{y}}^{*} + {\mathbf{m}}^{\prime } PEV{\mathbf{m}}. \\ \end{aligned}$$

The expectation of the log-likelihood ratio thus becomes:

$$\begin{aligned} {\text{Log}}\left( {LR} \right) = & \,\frac{1}{2}\log \left( \lambda \right) - \frac{1}{2}\log \left( {{\mathbf{m}}^{\prime } {\mathbf{m}} + \lambda } \right) \\ & + \frac{1}{2}\frac{{{\mathbf{y}}^{*\prime } {\mathbf{mm}}^{\prime } {\mathbf{y}}^{*} + {\mathbf{m}}^{\prime } PEV{\mathbf{m}}}}{{\left( {{\mathbf{m}}^{\prime } {\mathbf{m}} + \lambda } \right)\sigma_{e}^{2} }}. \\ \end{aligned}$$
